# De novo implantation vs. upgrade cardiac resynchronization therapy: a systematic review and meta-analysis

**DOI:** 10.1007/s10741-017-9652-1

**Published:** 2017-10-19

**Authors:** Annamaria Kosztin, Mate Vamos, Daniel Aradi, Walter Richard Schwertner, Attila Kovacs, Klaudia Vivien Nagy, Endre Zima, Laszlo Geller, Gabor Zoltan Duray, Valentina Kutyifa, Bela Merkely

**Affiliations:** 1 Heart and Vascular Center, Semmelweis University, 68 Városmajor Street, Budapest, 1122 Hungary; 2 University Hospital Frankfurt—Goethe University, Frankfurt am Main, Germany; 3 Medical Centre—Hungarian Defence Forces, Budapest, Hungary; 4 Heart Center, Balatonfüred, Hungary; 5 University of Rochester, Medical Center, Rochester, NY USA

**Keywords:** Heart failure, Cardiac resynchronization therapy, Mortality, Meta-analyses, CRT upgrade, De novo CRT

## Abstract

Patients with conventional pacemakers or implanted defibrillators are often considered for cardiac resynchronization therapy (CRT). Our aim was to summarize the available evidences regarding the clinical benefits of upgrade procedures. A systematic literature search was performed from studies published between 2006 and 2017 in order to compare the outcome of CRT upgrade vs. de novo implantations. Outcome data on all-cause mortality, heart failure events, New York Heart Association (NYHA) Class, QRS narrowing and echocardiographic parameters were analysed. A total of 16 reports were analysed comprising 489,568 CRT recipients, of whom 468,205 patients underwent de novo and 21,363 upgrade procedures. All-cause mortality was similar after CRT upgrade compared to de novo implantations (RR 1.19, 95% CI 0.88–1.60, *p* = 0.27). The risk of heart failure was also similar in both groups (RR 0.96, 95% CI 0.70–1.32, *p* = 0.81). There was no significant difference in clinical response after CRT upgrade compared to de novo implantations in terms of improvement in left ventricular ejection fraction (ΔEF de novo − 6.85% vs. upgrade − 9.35%; *p* = 0.235), NYHA class (ΔNYHA de novo − 0.74 vs. upgrade − 0.70; *p* = 0.737) and QRS narrowing (ΔQRS de novo − 9.6 ms vs. upgrade − 29.5 ms; *p* = 0.485). Our systematic review and meta-analysis of currently available studies reports that CRT upgrade is associated with similar risk for all-cause mortality compared to de novo resynchronization therapy. Benefits on reverse remodelling and functional capacity improved similarly in both groups suggesting that CRT upgrade may be safely and effectively offered in routine practice. Clinical Trial Registration: Prospero Database—CRD42016043747

**Electronic supplementary material:**

The online version of this article (10.1007/s10741-017-9652-1) contains supplementary material, which is available to authorized users.

## Introduction

Cardiac resynchronization therapy (CRT) has been shown to improve cardiac function, symptoms and hospitalization and reduce all-cause mortality in heart failure patients with prolonged QRS and reduced ejection fraction [1]. Since chronic right ventricular pacing could be deleterious by increasing the risk of heart failure, all-cause mortality and atrial fibrillation [2, 3], patients implanted with conventional pacemaker or implantable cardioverter defibrillator (ICD) systems are often considered for upgrading to CRT.

Recent studies suggested that patients with typical left bundle branch block (LBBB) ECG morphology derive the most benefit from CRT [4, 5]. Although right ventricular pacing could trigger similar ventricular dyssynchrony to LBBB, data are scarce regarding the benefits of upgrading to CRT in patients with previously implanted cardiac pacemaker or ICD systems.

The latest ESC guidelines on cardiac pacing and resynchronization therapy recommends CRT upgrade as a class I indication (level B) for symptomatic patients (New York Heart Association (NYHA) III–IVa) with low ejection fraction (LVEF ≤ 35%) [6]; however, the most recent European heart failure guidelines restrict this indication as a class IIb (level B) [7], due to lack of randomized clinical data available. In contrast, ACC guidelines focus mostly on the percentage of right ventricular pacing rather than symptoms or functional status [8].

While we are awaiting further data of prospective clinical trials on the effects of CRT upgrade on left ventricular reverse remodelling and clinical outcomes, we need more data for routine clinical practice. Therefore, we aimed to provide a detailed analysis of the available evidence comparing clinical outcomes and long-term survival between CRT upgrade and de novo implantations.

## Methods

### Study selection

This systematic review was performed according to the PRISMA statement [9], and a predefined review protocol was published in the PROSPERO database under the registration number of CRD42016043747 [10]. A comprehensive search of PubMed, ResearchGate and GoogleScholar databases was performed from January 2006 to March 2017 focusing on full-sized, peer-reviewed, English language papers reporting data on patient outcomes after upgrade CRT vs. de novo implantations as a comparator group. In order to identify all potentially relevant articles, the search was performed by using the terms of (1) “upgrade” AND “CRT” and (2) “upgrade” AND “cardiac resynchronisation therapy”. The search was also extended by using the name of the most frequently cited authors of the identified studies. In addition, references of relevant review articles were also searched to find appropriate manuscripts.

Potentially relevant articles were evaluated by three independent reviewers (A.K., M.V., R.S.), and additional manuscripts were retrieved that either reviewer felt were potentially relevant. According to our review protocol, studies were accepted for analysis if (i) including heart failure patients with reduced ejection fraction (HFrEF) with de novo and upgrade CRT implantations, (ii) reporting all-cause mortality data or heart failure events and (iii) reporting echocardiographic (i.e. LVEF, end-diastolic volume (EDV)) or clinical (NYHA class) or ECG (QRS width) parameters of reverse remodelling (Supplementary Table 1). Heart failure events were defined as hospitalization due to progression of heart failure. Corresponding authors were contacted for unpublished information and permission in the case of missing relevant data sets. In order to evaluate the heterogeneity of patients who were enrolled into each therapy groups, the most important baseline clinical characteristics were collected and compared. Data on procedure-related complications were also collected if available.

### Statistical analysis

All statistical analyses were conducted utilizing Comprehensive Meta-Analysis 3.3 (Biostat, Inc., USA) and GraphPad Prism Software Version 7 (GraphPad Prism Inc., San Diego, CA, USA). Heterogeneity between individual trial estimates was assessed using the *Q* statistic and *I* ^2^ statistic [11]. Since there was significant heterogeneity in the design and patient characteristics of the included studies, it was assumed that the true effect size varies from one study to the other, and hence, the random-effect model was used [12]. As a principal yet conservative measurement of the effect size (i.e. all-cause mortality), we calculated risk ratios (RRs) along with a 95% upper and lower confidence interval (CI) and compared the two therapy groups as case-control models. Additionally, meta-analysis was performed for publications where crude and/or adjusted hazard ratios (HRs) were also available. Sensitivity analysis with the inclusion of prospective studies only was performed. Forest plots were constructed showing the individual trials with the pooled estimates. Publication bias was assessed using the funnel plot, the trim and fill method of Duval and Tweedie [13] and an adjusted rank correlation test according to Begg and Mazumdar [14]. Since we did not have access to individual patient data from all studies reviewed, the median of delta values for LVEF, EDV, NYHA and QRS was calculated and compared between the two patient groups by using the Mann-Whitney *U* test. Methodological quality of all studies was assessed using the methodological index for non-randomized studies (MINORS) [15, 16]. Studies were defined to be low, moderate and high-quality studies based on their MINORS scores of < 8, < 16 and ≥ 16 points (Supplementary Table 2).

## Results

### Study characteristics

**Fig. 1:**
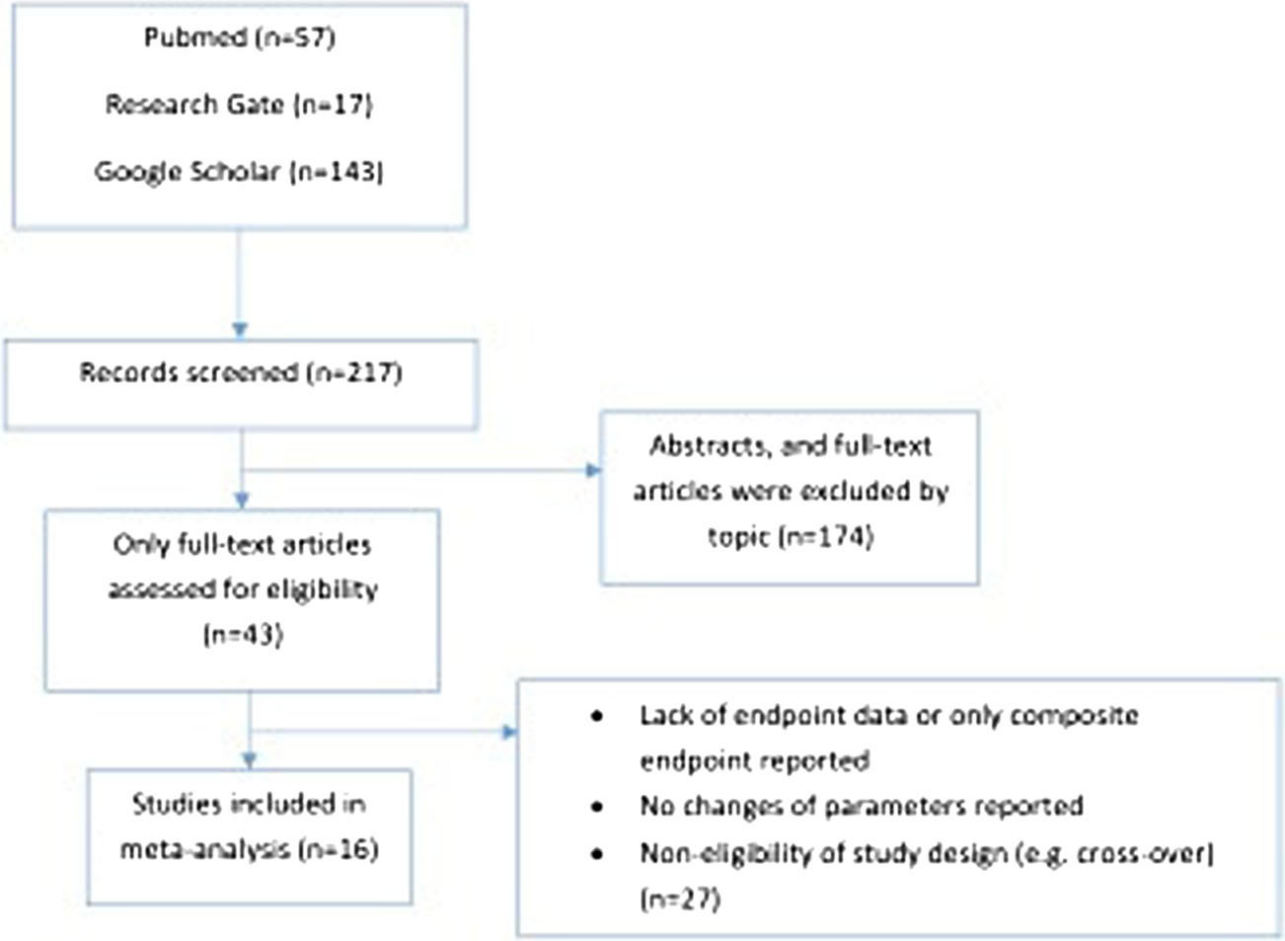
Flow chart of searching for publications

A total of 16 reports were selected for the current analysis comprising 489,568 CRT recipients, of whom 468,205 patients had de novo resynchronization therapy and 21,363 patients underwent an upgrade procedure (Fig. 1). The characteristics of all included studies are shown in Table 1. None of the identified studies was a randomized, controlled trial. Most of them were observational, retrospective [17–28] or observational prospective [29–32] cohort studies. The vast majority were single-centre observations [17, 19–21, 23–26, 29, 30] with the exception of four dual/multicentre studies [18, 22, 28, 32] and two based on high volume registries (European survey [27] and United States National Database [28]). Four [26, 29, 31, 32] from the 16 studies proved to be high-quality reports (average MINORS score 11.4, Supplementary Table 2).

**Table 1:** Characteristics of included studies

Study, year	Design	Number of patients		Follow-up (median of short/long term)	Endpoints	Type of devices before upgrade		% of ventricular pacing before upgrade	Study quality—MINORS score
		Total	De novo	Upgrade					
Marai et al. [30], 2006	Single-centre, prospective observational cohort	98	73	25	3 months	ΔEF ΔNYHA (6 MWT)	PMs (VVI/VDD/DDD)	PM-dependent patients with constant RVAP for 4.7 ± 2.5 years	Moderate
Witte et al. [25], 2006	Single-centre, retospective observational cohort	71	39	32	3 months	ΔEF ΔEDV ΔQRS (dyssychrony parameters)	PMs (further details are NA)	> 50%	Moderate
Duray et al. [29], 2008	Single-centre, prospective observational cohort	79	61	18	6 months	All-cause mortality (procedural parameters, NYHA/LVEF/NT-proBNP)	PMs/ICDs	NA	High
Nagele et al. [22], 2008	Multicentre retrospective, population-based cohort	328	221	107	12/30 months	All-cause mortality ΔEF ΔNYHA ΔQRS (QoL, peak Vo2, dyssynchrony parameters)	81% DDD 19% VVI	96 ± 4%	Moderate
Foley et al. [17], 2009	Single-centre, retrospective observational cohort	394	336	58	12 / 25 months	ΔEF ΔEDV ΔNYHA All-cause mortality, (CV death or HF hospitalization, 6 MTW, QoL)	VVI or DDD	81 ± 31.0%	Moderate
Wokhlu et al. [26], 2009	Single-centre, retrospective observational cohort	505	338	167	7.1/31.2 months	All-cause mortality ΔEF ΔEDV ΔNYHA	54.5% ICD 45.5% PM	< 40% in 25% of pts 40–80% in 21% of pts > 80% in 54% of pts	High
Frohlich et al. [18], 2010	Multicentre retrospective population-based cohort	172	102	70	21 months	ΔEF ΔQRS (NYHA)	NA	> 50% for at least 6 months before including	Moderate
Paparella et al. [23], 2010	Single-centre, retrospective population based cohort	82	43	39	1.3 and every 6 months thereafter /35 months	Heart failure events ΔEF ΔEDV ΔNYHA ΔQRS (6 MWT, dyssynchrony parameters, MR)	31% VVI 43% DDD 25% VDD	91 ± 7%	Moderate
Kabutoya et al. [20], 2010	Single-centre, retrospective observational cohort	48	33	15	6 months	ΔEF (LV dP/dt)	47% PM 53% ICD	94 ± 11%	Moderate
Bogale et al. [27], 2011	Multicenter, survey registry	2367	1489	601	12 months	All-cause mortality Heart failure events (NYHA, QRS, procedural parameters)	30.1% PM 69.9% ICD	62% paced rhythm at inclusion, no further details	Moderate
Gage et al. [19], 2014	Single-centre, retrospective observational cohort	655	465	190	12 months	All-cause mortality Heart failure events ΔEF ΔEDV (dyssynchrony parameters, MR, RV dysfunction)	58% PM 42% ICD	> 40%	Moderate
Tayal et al. [31], 2016	Single-centre, prospective observational cohort	135	85	50	6/48 months	All-cause mortality ΔEF (MR, global long. strain)	PMs	> 40%	High
Horst et al. [24], 2016	Single-centre, retrospective observational cohort	268	134	134	12 months	All-cause mortality (procedural parameters)	PMs and ICDs; 60% DDD, 40% VVI	NA	Moderate
Lipar et al. [21], 2016	Single-centre, retrospective observational cohort	281	165	116	10 months	All-cause mortality Heart failure events ΔEF ΔNYHA ΔQRS	49% DDD PM, 22% DDD-ICD, 18% VVI, 12% VVI-ICD	< 40% in 13% of pts 40–80% in 16% of pts > 80% in 71% of pts	Moderate
Vamos et al. [32], 2017	Multicentre, prospective observational cohort	552	375	177	37 months	All-cause mortality ΔEF ΔNYHA	PMs/ICDs	NA	High
Cheung et al. [28], 2017	Multicentre, retrospective observational cohort	483,810	464,246	19,564	NA	All-cause mortality, procedural parameters	PMs/ICDs	NA	High

*EDV* end-diastolic volume, *EF* left ventricular ejection farction, *ICD* implantable cardiac defibrillator, *PM* pacemaker, *DDD-PM/ICD* dual-chamber pacemaker or ICD, *VVI-PM/ICD* single-chamber ventricular pacemaker or ICD, *Pts* patients, *NYHA* New York Heart Association Class, *MR* mitral regurgitation, *RVAP* right ventricular apical pacing

The most important published patient characteristics of the included studies, such as age, gender, aetiology, baseline QRS duration (paced in upgrade, intrinsic in de novo groups), baseline NYHA functional class, baseline left ventricular ejection fraction and dimensions are summarized in Supplementary Table 3. In summary, the mean ejection fraction was by definition lower than 35% in all studies, and there were no significant differences between the de novo and upgrade groups in most of the individual studies. Most of the trials enrolled patients with severe symptoms (NYHA III–IVa); a smaller extent of the studies investigated patients without depicting functional class. More than 50% of the studies found significant differences in the following baseline parameters between the two patient groups: age, atrial fibrillation and QRS duration. In the upgrade group, patients were generally older, more likely to have atrial fibrillation and they had wider (paced) QRS.

### All-cause mortality and heart failure events

**Fig. 2:**
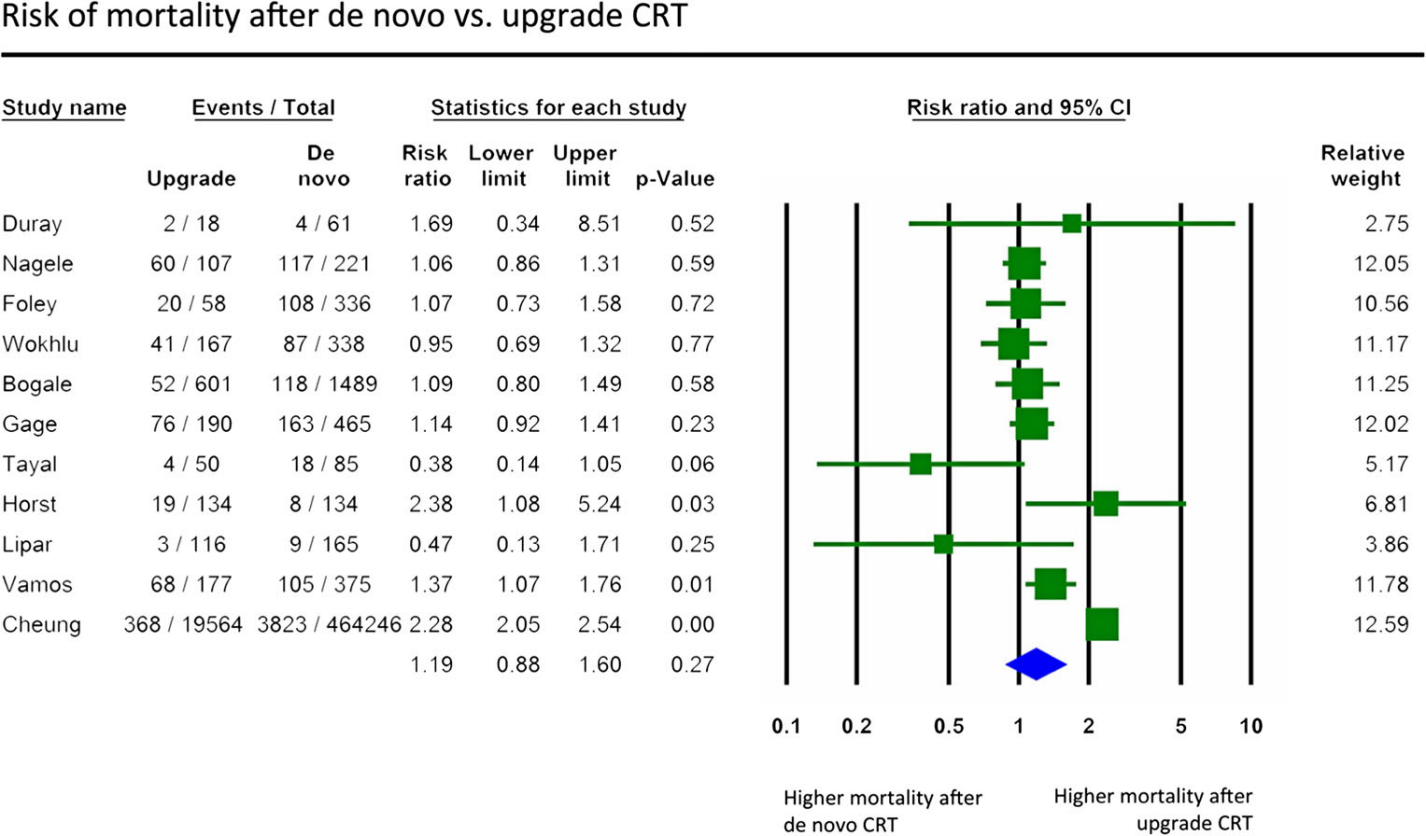
Risk of all-cause mortality (risk ratio) after de novo vs. upgrade CRT

Crude mortality rates were available in 489,197 patients from 11 studies [17, 19, 21, 22, 24, 26–29, 31, 32], while unadjusted or adjusted hazard ratios were available for 1734 and 1229 patients in 4 [19, 26, 31, 32] and 3 [19, 31, 32] studies, respectively. All-cause mortality did not differ following upgrade compared to de novo implantations (RR 1.19, 95% CI 0.88 to 1.60, *p* = 0.27, *I* ^2^ = 90.1%, Fig. 2). Pooled analyses of the unadjusted or adjusted hazard ratios revealed similar findings (crude HR 1.07, 95% CI 0.72 to 1.57, *p* = 0.74, *I* ^2^ = 73.6%, Supplementary Fig. 1a) (adjusted HR 0.81, 95% CI 0.36 to 1.81, *p* = 0.61, *I* ^2^ = 88.5%, Supplementary Fig. 1b).

When only prospective studies were analysed, no differences were found between the two groups (RR 1.10, 95% CI 0.76 to 1.60, *p* = 0.60, *I* ^2^ = 54.0%, Supplementary Fig. 1c).

**Fig. 3:**
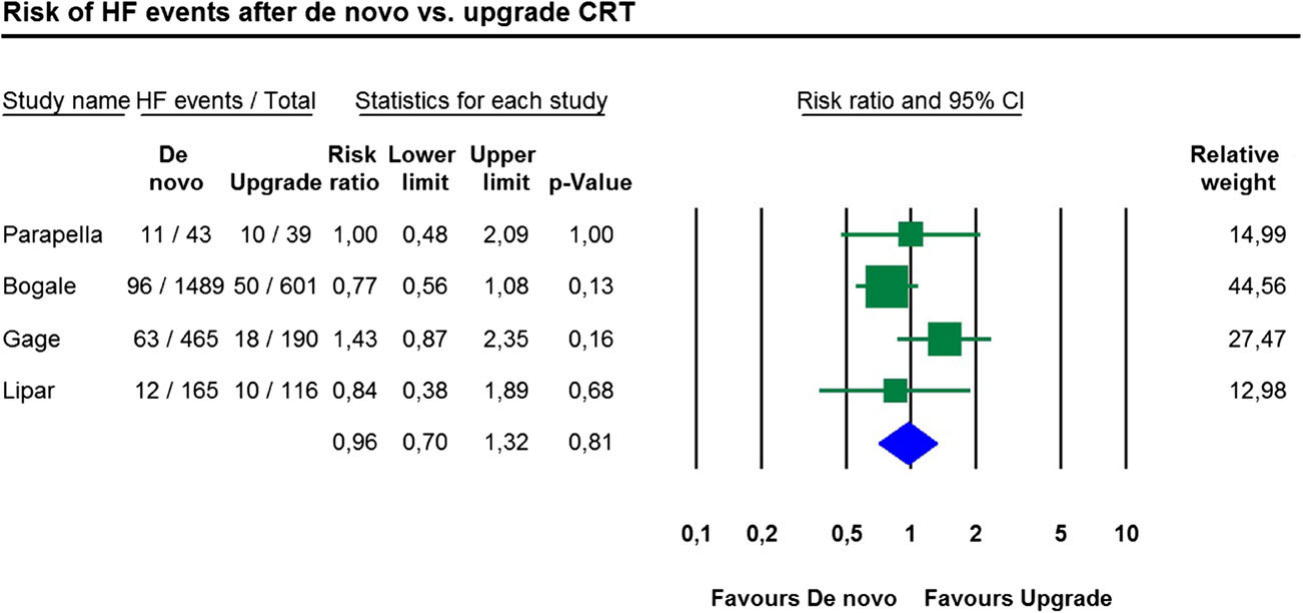
Risk of heart failure events after de novo vs. upgrade CRT

In studies providing appropriate information, the unadjusted risk of heart failure was also similar in de novo and upgrade CRT groups (RR 0.96, 95% CI 0.70 to 1.32, *p* = 0.81, *I* ^2^ = 28.0%, Fig. 3).

### Left ventricular reverse remodelling, clinical improvement

**Fig. 4:**
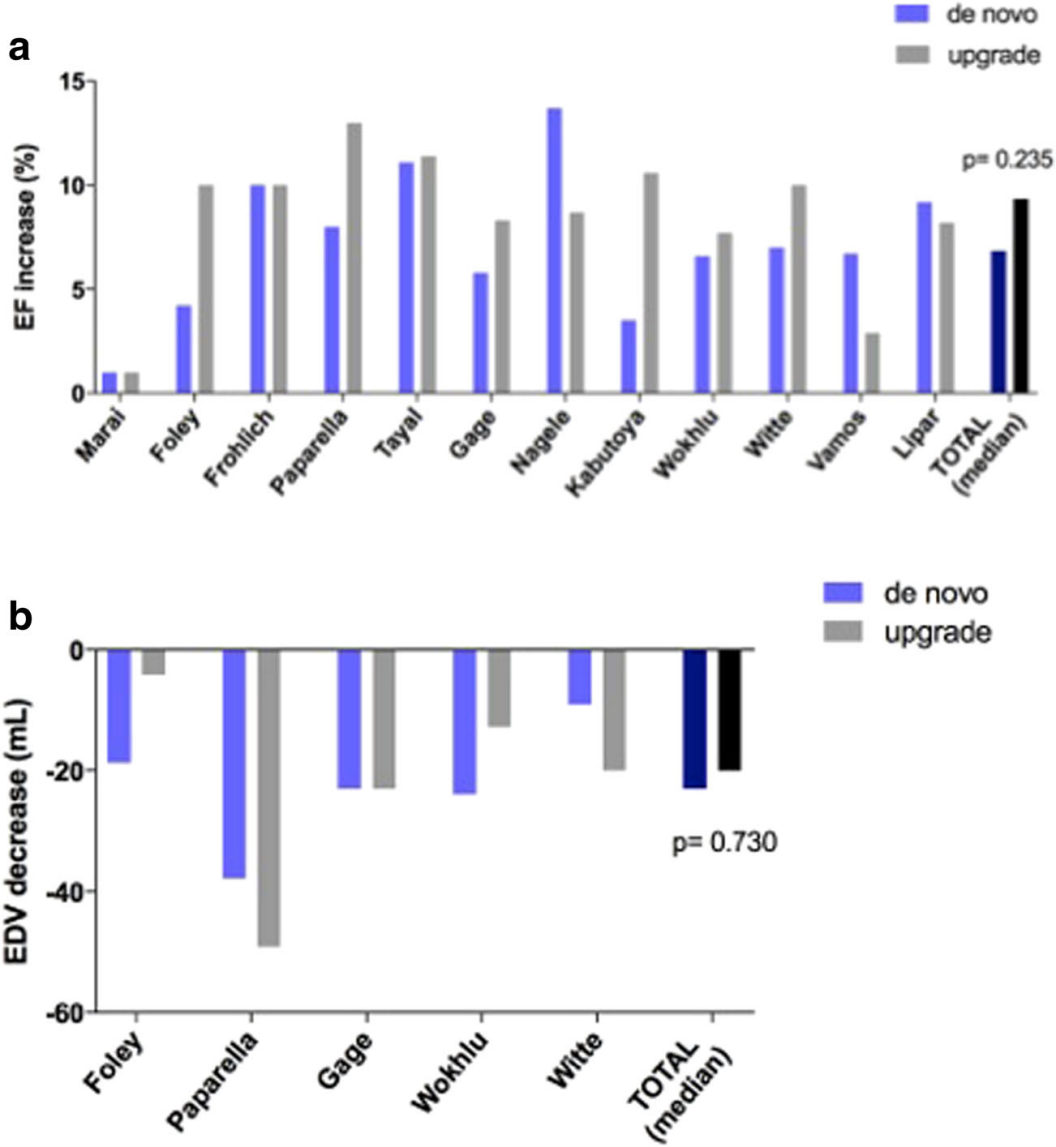
**a** Change in ejection fraction after de novo vs. upgrade CRT. **b** Change in end-diastolic volume after de novo vs. upgrade CRT

**Fig. 5:**
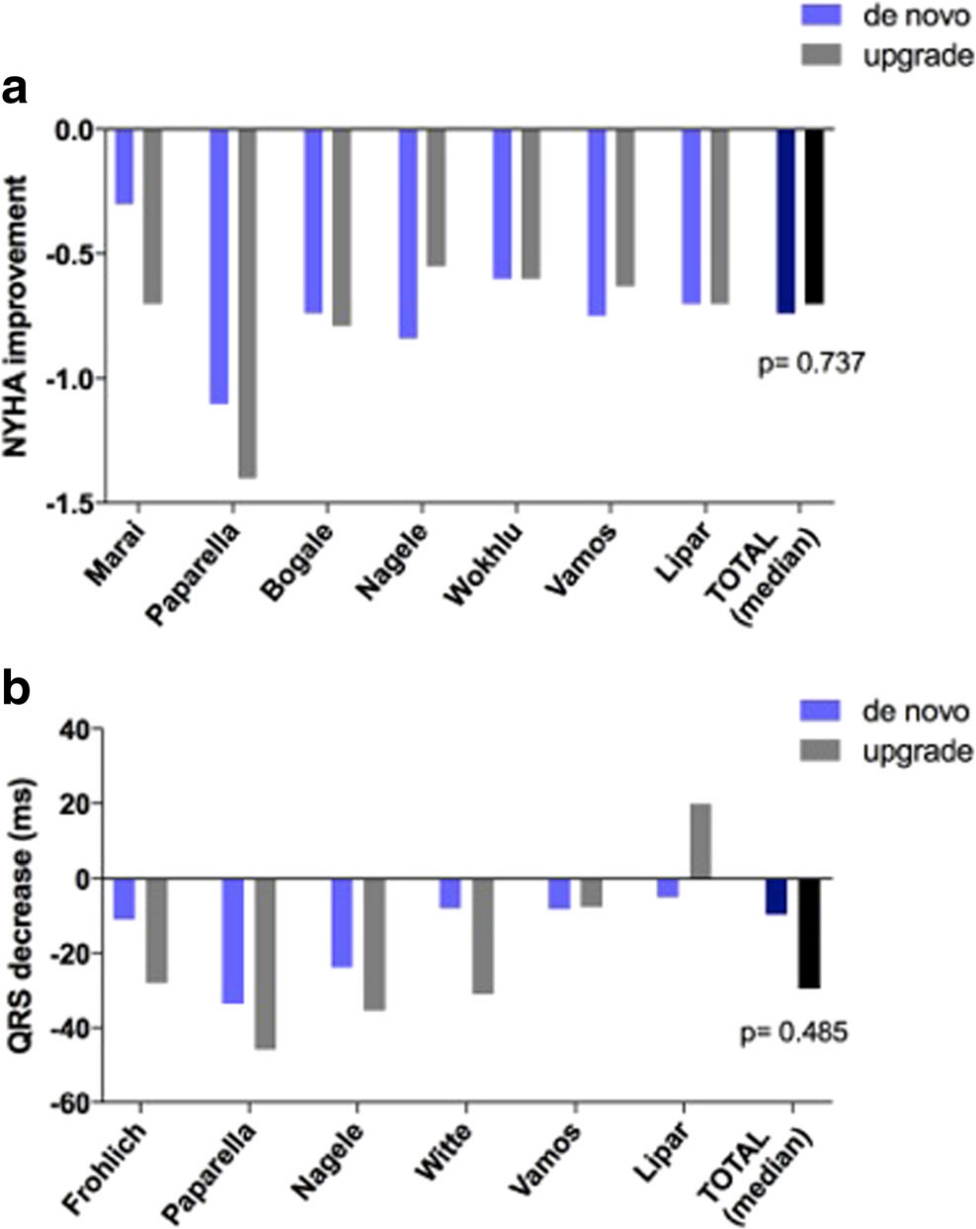
**a** Change in NYHA functional class after de novo vs. upgrade CRT. **b** Change in QRS duration after de novo vs. upgrade CRT

The extent of reverse remodelling in terms of improvement in left ventricular ejection fraction and end-diastolic volume was similar in the two patient groups (ΔEF de novo − 6.85% vs. upgrade − 9.35%, *p* = 0.235; ΔEDV de novo − 23.0 vs. upgrade − 20.0 ml; *p* = 0.730) (Fig. 4a, b). Regarding symptoms, change in NYHA functional class was also comparable after de novo CRT implantation and upgrade procedures (ΔNYHA de novo − 0.74 vs. upgrade − 0.70 class; *p* = 0.737) (Fig. 5). When QRS narrowing was compared, no significant difference was found between the two patient groups (ΔQRS de novo − 9.6 vs. upgrade − 29.5 ms; *p* = 0.485) (Fig. 5b).

### System-related complications

Based on four studies [24, 27–29], where detailed analyses regarding system-related complications were published, fluoroscopic time [27], the rate of phrenic nerve stimulation [24], cardiac perforation, pneumothorax and lead dislocation [28] showed significant difference between the two patient groups (Table 2). In the largest database [28], the most severe complications such as lead revision, pneumothorax or perforation were observed more frequently in the upgrade group.

**Table 2:**
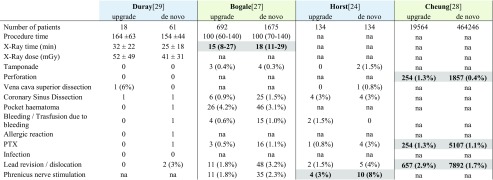
Complications during de novo CRT vs. upgrade CRT implantations

Parameters with significant difference in the original reports are highlighted with bold verbatim

Another prospective, multicentre registry which was designed to demonstrate complication rates in patients with 6 months after pacemaker or ICD replacement has to be mentioned; however due to its study design, it was not eligible for including in the current analyses [33]. In this registry [33], 713 patients were upgraded, and the most frequent major complication was lead dislodgement or malfunction observed in 7.9% of patients, while 1.5% experienced haematoma and 0.8% infection in the first 6 months after the procedure.

### Publication bias

According to the rank correlation test of Begg and Mazumdar, there was no evidence of significant publication bias (mortality RR: *τ* = − 0.236, *p* = 0.312; mortality RR in prospective trials: *τ* = − 0.167, *p* = 0.734; mortality crude HR: *τ* = − 0.167, *p* = 0.734; mortality adjusted HR: *τ* = 0.333, *p* = 0.602; HF RR: *τ* = 0, *p* = 1.000). Furthermore, corresponding to the Duval and Tweedie’s trim and fill input method, there was no evidence that publication bias would significantly impact on the overall effect sizes observed (Supplementary Figs. 2–6).

## Discussion

### Main findings

This systematic review of 16 studies comparing data in approximately 500,000 patients undergoing de novo or upgrade CRT implantations revealed no significant difference in all-cause mortality or heart failure events between the two patient groups. Also, no significant differences were found in changes of echocardiographic parameters of reverse remodelling (EF, EDV). Functional changes (i.e. improvement of NYHA functional class) and narrowing of QRS were also similar, suggesting that adding left ventricular pacing in patients with prior cardiac devices may be a safe and feasible procedure with similar clinical benefits as de novo implantations.

### Patient population referred to biventricular upgrade

Biventricular upgrade affects roughly 5–10% of patients who underwent ICD or pacemaker implantation [33, 34]. Due to the right ventricular (RV) pacing-induced dyssynchrony, patients with a high percentage of RV pacing are at high risk of adverse clinical outcomes [2, 3] and could become candidates for CRT upgrade. Wilkoff et al. demonstrated in the DAVID trial that the percent of RV pacing correlated with the composite of death or rehospitalizations for HF in ICD recipients with a high rate of DDD pacing compared to patients with the VVI 40/min programming [3]. In addition, echocardiographic and functional parameters (6-min walk test, symptoms) may worsen even in patients with previously preserved ejection fraction [35, 36] or mild heart failure [37] after frequent RV pacing. In the BLOCK-HF [37] trial, patients with atrioventricular (AV) block, mild symptoms (NYHA II–III) and HFmrEF or HFrEF (EF < 50%, the baseline mean EF = 45%) received a CRT device and were randomly assigned to standard right ventricular or biventricular pacing. The primary endpoint (composite of all-cause mortality, HF events, or ≥ 15% increase in LVESV index) occurred in 190 of 342 patients (55.6%) in the RV pacing group, compared to 160 of 349 patients (45.8%) in the CRT group, which first demonstrated the superiority of biventricular pacing over RV pacing in pacemaker-dependent patients.

According to these lines of evidences and considerations, it seems reasonable upgrading to CRT in HF patients with previously implanted cardiac devices and a high percentage of right ventricular pacing. On the other hand, upgrade procedures may be associated with higher surgical risk, such as venous access issues, the risk of damage or extraction of previously implanted leads, higher infection rates and longer procedure times [33, 38], that all together may significantly compromise the success of LV pacing.

It should be also noted that aetiology or the cause of decreased ejection fraction might be different in upgrade vs. de novo CRT groups. Regarding the aetiology, similar percentage of ischemic and non-ischemic heart disease was reported in most of the included studies; however, the baseline QRS was wider (paced QRS), and patients were older and had more often atrial fibrillation in the upgrade group.

### Evidence supporting CRT upgrade

The current guideline recommendations are mainly based on some non-randomized, observational prospective “upgrade vs. de novo” studies, which are included in the current analysis [17, 18, 23, 27, 30]. In addition, small observational retrospective [39–45] and cross-over [46–49] trials are also referred in the ESC guidelines with a low number of patients.

In most of these trials, only soft endpoints, such as NYHA functional class, 6-min walk test, quality of life or echocardiographic parameters were analysed. Summarizing the most frequently investigated clinical parameters, such as change in NYHA functional class, decrease in QRS duration, changes of left ventricular ejection fraction and end-diastolic volume, no significant differences were observed between the de novo and upgrade groups in our analysis.

Data regarding long-term mortality were reported only in a few prior trials [17, 19, 21, 22, 24, 26–29, 31, 32]. The largest report from these was the European Cardiac Resynchronization Survey [27] from 2011 comprising 1489 de novo and 601 upgrade CRT patients. Total mortality at 1 year was low and similar in both groups (8.6 vs. 7.9%, *p* = 0.57). Although this registry showed representative data about mortality rates with high number of enrolled patients, there are a huge number of potential confounders that may have biased the overall results. Therefore, trials with adjusted analyses are essential to control baseline differences to better assess the effects of CRT upgrade on long-term survival. In the current meta-analysis, three observational studies with adjusted all-cause mortality endpoints were included. Tayal et al. compared 85 patients who underwent de novo CRT implantation and 50 patients with CRT upgrade [31]. During the 4 years of follow-up time, patients with prior right ventricular pacing had a significantly lower risk of fatal events than patients with de novo CRT implantation (adjusted HR 0.25, 95% CI 0.07–0.88, *P* = 0.03). Gage et al. compared 190 patients with prior high percentage of right ventricular pacing (> 40%) to 465 non-paced patients who underwent CRT implantation [19]. During the median follow-up of 4.2 years, upgrade patients tended to have better outcomes in terms of all-cause mortality (adjusted HR 0.73; 95% CI 0.53–1.01; *p* = 0.055). In contrast, Vamos et al. recently reported a higher risk for mortality in the upgrade group when compared to de novo implantation in 552 patients [32]. In this multicentre study with a mean follow-up of 37 months, patients who underwent CRT upgrade had a significantly higher risk of all-cause mortality compared to patients with de novo implantations even after adjusting for potential confounders with multivariate Cox regression analysis (adjusted HR 1.68, 95% CI 1.20–2.34, *p* = 0.002) and after applying propensity score matching (PS-adjusted HR 1.79, 95% CI 1.08–2.95, *p* = 0.023). Summarizing all these results in our meta-analysis, a similar long-term survival was found between the two patient groups. However, heterogeneities in the results of adjusted studies largely emphasize that randomized controlled trials are needed to objectively clarify this clinical dilemma.

Besides the aforementioned publications until 2016, there is another manuscript released in 2017 which has to be discussed more in details. Cheung et al. recently published a unique data using the US National Database between 2003 and 2013 including 19,546 patients who underwent CRT upgrade vs. 464,246 patients after de novo CRT implantations [28], which was also included in the current analysis. This study found that patients with de novo CRT implantation were older, had more frequent third-degree AV block, LBBB or other comorbidities such as renal failure or ischemic heart disease. The rate of in-hospital mortality of patients undergoing CRT upgrade was significantly higher than in the de novo group (1.9 vs. 0.8%; *p* < 0.001). Regarding complications, significantly higher rate of pneumothorax, lead revision or perforation were observed in the upgrade group. These results are somewhat different from other trials included in our meta-analysis, where patients undergoing CRT upgrade were generally older and had more frequent atrial fibrillation. Despite potential differences in the lengths of follow-up and baseline patient characteristics, other studies did not reveal a higher risk for mortality, such as the twofold higher risk in the US cohort. This pronounced in-hospital mortality rate might be derived from a de-identified, code-based selection of their database which may not have been representative for the total patient cohort dedicated for CRT upgrade.

Despite the current detailed review and meta-analysis of the available clinical evidence, several questions remain unanswered. Most striking from these include which populations may derive the largest benefits from upgrading and what is the optimal timing for such procedures.

The ongoing BUDAPEST-CRT Upgrade study (NCT02270840) was designed to evaluate the efficacy and safety of CRT upgrade from conventional PM or ICD systems [50]. In this prospective, randomized, multicentre clinical trial symptomatic heart failure patients (NYHA II–IVa) with low ejection fraction (EF ≤ 35%), intermittent (≥ 20%) or permanent right ventricular pacing and wide paced QRS (≥ 150 ms) are randomized to CRT-D or ICD. Based on the primary composite endpoint of all-cause mortality, heart failure events and less than 15% end-systolic volume reduction at 12-month follow-up, we will obtain more definite data on the risks and benefits of CRT upgrade procedures.

## Limitations

This meta-analysis shows all potential limitations of such a kind of analysis. Patients in the two groups were not randomly allocated; all included studies were either retrospective studies with historical controls or prospective observational data collections; thus, a residual selection bias could not be excluded. There are remaining clinical issues that have obviously affected the decision-making on upgrading but could not be collected and analysed in a systematic fashion, such as the possibility to avoid RV stimulation by programming appropriately, the patency of the venous system, need for generator or lead replacement due to battery or lead issues and end-stage renal disease on dialysis or other severe comorbidities including age. Second, the comparison of these two groups is partly confusing, while the aetiology of dyssynchrony may be different. In recipients of “de novo” CRT, other underlying cardiac pathophysiological conditions in addition to the initial dyssynchrony may be present, whereas in the upgrade group, patients were initially implanted with a pacemaker for a bradycardia indication. Third, we did not have access to individual patient-level data precluding us from calculating adjusted hazard ratios for all the included studies. Finally, the length of follow-up was also heterogeneous in the included reports. However, so far, this is the largest available comprehensive evidence in this respect, and sensitivity analysis from adjusted results corroborated our initial findings.

## Conclusions

Our systematic review and meta-analysis of currently available studies reports that CRT upgrade is associated with similar risk for all-cause mortality compared to de novo resynchronization therapy. Benefits on reverse remodelling and functional capacity improved similarly in both groups suggesting that CRT upgrade may be safely and effectively offered in routine practice. These results should be confirmed in further randomized clinical trials.

## Electronic supplementary material

ESM 1 (DOCX 4.53 mb)

## Abbreviations

CI: Confidence interval
CRT: Cardiac resynchronization therapy
EDV: End-diastolic volume
ESV: End-systolic volume
HF: Heart failure
HFrEF: Heart failure with reduced ejection fraction
HFmrEF: Heart failure with mid-range ejection fraction
HR: Hazard ratio
LVEF: Left ventricular ejection fraction
ICD: Implantable cardioverter defibrillator
LBBB: Left bundle branch block
NYHA: New York Heart Association Functional Class
RR: Risk ratio
VF: Ventricular fibrillation
