# Supplementary material for: De novo implantation vs. upgrade cardiac resynchronization therapy: a systematic review and meta-analysis
Source: Heart Fail Rev. 2017 Oct 19;23(1):15–26. doi: 10.1007/s10741-017-9652-1 (PMC5756552; doi:10.1007/s10741-017-9652-1)

**De novo implantation vs. upgrade cardiac resynchronization therapy: a systematic review and meta-analysis**

**Supplementary material**

Supplementary Table 1: Searching methodology and eligibility criteria for the meta-analysis

| Eligibility criteria | | |
| --- | --- | --- |
| Criteria | Included | Excluded |
| Participants | HF pts with wide QRS, NYHA II - ambulantory IV and EF≤ 35% | No indication for CRT |
| Intervention | CRT upgrade | Unsuccessful LV lead implantation |
| Comparator | De novo CRT implantation | No comparator group |
| Primary Outcome | All-cause mortality | Only cause specific mortality data or composit endpoints provided |
| Secondary outcomes | HF events, changes in NYHA class, echocardiographic parameters of reverse remodeling, QRS narrowing, procedure related complications | NA |
| Study design | Randomized controlled trials  Non-randomized trials  Observational cohort studies | Case reports  Reviews  Meta-analyses |
| Languages | English | Any other languages |
| Publication status | Published or accepted full-text manuscripts | Abstracts, unpublished |

Supplementary Table 2: Results of the quality assessement with MINORS criteria

| **Study** | **aim of the study** | **inculsion of consecutive patients** | **prospective data collection** | **appropriate endpoint to the aim of the study** | **unbaised evaluation of endpoints** | **follow-up period appropriate to the endpoint** | **loss to follow-up no more than 5%** | **comparable control group** | **Contemporary groups** | **baseline equivalence of groups** | **prospective calculation of the sample size** | **use of adequate statistical analysis** | **MINORS SCORE** |
| --- | --- | --- | --- | --- | --- | --- | --- | --- | --- | --- | --- | --- | --- |
| **Marai** | 1,5 | 2 | 1 | 1 | 1 | 0,5 | 0 | 1 | 1,5 | 1 | 0,5 | 2 | 13 |
| **Foley** | 2 | 1 | 1 | 1,5 | 1 | 1,5 | 1 | 1 | 1,5 | 1 | 0,5 | 2 | 15 |
| **Frohlich** | 1,5 | 2 | 0 | 1,5 | 1 | 1 | 0 | 1,5 | 1 | 1 | 0 | 2 | 12,5 |
| **Parapella** | 1,5 | 0,5 | 1 | 1 | 1 | 1 | 1,5 | 1 | 1,5 | 1 | 0,5 | 2 | 13,5 |
| **Bogale** | 2 | 0,5 | 0 | 2 | 2 | 2 | 0,5 | 1 | 2 | 1 | 0 | 2 | 15 |
| **Duray** | 1,5 | 1,5 | 1 | 1,5 | 2 | 0 | 1,5 | 2 | 1,5 | 2 | 0,5 | 2 | 17 |
| **Tayal** | 1 | 2 | 1 | 1,5 | 1 | 2 | 1,5 | 1 | 1,5 | 1 | 0,5 | 2 | 16 |
| **Gage** | 1 | 1,5 | 1 | 1,5 | 1 | 1 | 0,5 | 1 | 1,5 | 1 | 0,5 | 2 | 13,5 |
| **Horst** | 1,5 | 0 | 0 | 1,5 | 2 | 2 | 2 | 1,5 | 1 | 1 | 0 | 2 | 14,5 |
| **Nagele** | 2 | 1,5 | 0,5 | 1 | 1 | 1,5 | 0 | 1,5 | 1,5 | 1 | 0,5 | 2 | 14 |
| **Kabutoya** | 1,5 | 2 | 1 | 1,5 | 1 | 0,5 | 0 | 1 | 2 | 1 | 0,5 | 2 | 14 |
| **Wokhlu** | 2 | 2 | 1 | 2 | 1 | 1 | 1 | 1 | 2 | 1 | 0,5 | 2 | 16,5 |
| **Witte** | 1 | 1 | 1 | 1 | 1,5 | 1,5 | 0 | 1 | 1,5 | 1 | 0,5 | 2 | 13 |
| **Lipar** | 2 | 0 | 0 | 1,5 | 1 | 1 | 0 | 1 | 1 | 1 | 0 | 2 | 10,5 |
| **Vamos** | 2 | 2 | 1 | 1 | 2 | 2 | 1 | 2 | 2 | 0 | 0 | 2 | 17 |
| **Cheung** | 2 | 1 | 0 | 2 | 2 | 0 | 0 | 1 | 0 | 1 | 1 | 2 | 12 |

Supplementary Table 3: Differences in the baseline patient characteristics of the included studies. (Parameters with significant difference in the original reports are highlithed with bold verbatim)

|  | **Gender (male)** | | **Etiology (ischemic)** | | **Atrial Fibrillation** | | **Age (years)** | | **QRS (ms)** | | **NYHA** | | **EF (%)** | | **LV dimensions**  **(EDV / EDD)** | |
| --- | --- | --- | --- | --- | --- | --- | --- | --- | --- | --- | --- | --- | --- | --- | --- | --- |
| **Study, Year** | De novo | Upgrade | De novo | Upgrade | De novo | Upgrade | De novo | Upgrade | De novo | Upgrade | De novo | Upgrade | De novo | Upgrade | De novo | Upgrade |
| Marai et al., 2006 | 64 (88%) | 20 (80%) | 65 (89%) | 23 (92%) | **11 (15%)** | **8 (32%)** | 69 ± 9 | 72 ± 9 | **163 ± 30** | **203 ± 32** | 3.1 ± 0.6 | 3.2 ± .5 | 22 ± 5 | 23 ± 9 | 67 ± 10 mm | 64 ± 6 mm |
| Witte et al., 2006 | NA | NA | 21 (54%) | 16 (50%) | **3 (8%)** | **17 (53%)** | 67± 2 | 70 ± 4 | **173 ± 4** | **207 ± 5** | 3.2 ± 0.5 | 3.3 ± 0.5 | 20 ± 1 | 20 ± 2 | 70 ± 2 mm | 70 ± 2 mm |
| Duray et al., 2008 | 50 (82%) | 13 (72%) | 30 (49%) | 8 (44%) | NA | NA | 63 ± 11 | 66 ± 10 | NA | NA | NYHA II: 33%  NYHA III-IV: 67% | NYHA II: 11%  NYHA III-IV: 89% | 22 ± 7 | 25 ± 9 | NA | NA |
| Nagele et al., 2008 | 80% | 92% | 53% | 49% | **14%** | **37%** | 68.4 ± 11 | 68.7 ± 15 | **168.3 ± 24** | **187.1 ± 28** | 3.1 | 3.1 | 26.4 ± 9 | 28.1 ± 6 | 63 ± 9 mm | 65 ± 13 mm |
| Foley et al., 2009 | 261 (78%) | 48 (83%) | 219 (65%) | 42 (72%) | NA | 14 (24%) | **68.7± 10.8** | **72.8 ± 11.4** | **150.9 ± 27.8** | **163.1 ± 32.3** | 3.26 ± 0.54 | 3.36 ± 0.55 | 23.2 ± 10.2 | 23.1 ± 10.7 | **253.5 ± 99.4 mL** | **212.5 ± 98.0 mL** |
| Wokhlu et al., 2009 | **253 (75%)** | **146 (87%)** | 204 (60%) | 110 (66%) | **87 (26%)** | **69 (41%)** | **67.7 ± 11.8** | **70.1 ± 10.3** | **158 ± 31** | **184 ± 32** | 3.0 ± 0.5 | 3.0 ± 0.5 | 23.1 ± 7.3 | 23.2 ± 7.2 | 235.6 ± 73.7 mL | 225.0 ± 69.6 mL |
| Frohlich et al., 2010 | 82 (80.4%) | 51 (72.9 %) | 41 (40.2%) | 36 (51.4%) | **32 (31.4%)** | **37 (52.9%)** | **61.0 (54–67)** | **66.5 (57.0–75.0)** | **154 (133–178)** | **184 (163–205)** | NYHA II: 20%  NYHA III: 75%  NYHA IV: 5% | NYHA II: 29%  NYHA III: 69%  NYHA IV:3% | 20.0 (16.8–29.0) | 24.0 (17.8–30.0) | 244 (175–305) mL | 219 (141–259 mL |
| Paparella et al., 2010 | 24 (56%) | 23 (59%) | 10 (23%) | 16 (41%) | NA | 8 (21%) | **71.5 ± 7.8** | **75.4 ± 5.8** | **172.1 ± 25.8** | **186.2 ± 22.1** | NYHA III: 55%  NYHA IV: 18% | NYHA III: 51%  NYHA IV: 30% | **26 ± 2** | **23 ± 7** | 214.5 ± 54.8 mL | 234.1 ± 48.4 mL |
| Kabutoya et al., 2010 | **79%** | **47%** | **33%** | **0%** | NA | NA | 65.4 ±11.2 | 68.3 ± 11.5 | **162 ± 25** | **189 ± 40** | NA | NA | **32 ± 12** | **30 ± 10** | NA | NA |
| Bogale et al., 2011 | NA | NA | **778 (49.7%)** | **339 (54.6%)** | **336 (20.3%)** | **208 (30.4%)** | **69 (62–76)** | **71 (64–77)** | **152 ± 28** | **171 ± 35** | NYHA I: 1.6%  NYHA II:20%  NYHAIII:70.1%  NYHA IV:8.3% | NYHA I: 0.8%  NYHA II:18.6%  NYHAIII:71.1%  NYHA IV:9.5% | **26 ± 8** | **28 ± 8** | NA | NA |
| Gage et al., 2014 | 68 | 75 | 58 | 58 | **13** | **37** | **69 ±12** | **73 ± 11** | **171 ± 28** | **152 ± 24** | NYHA III: 66% | NYHA III: 67% | 26.6 ± 6 | 26.7 ± 5 | **62 ± 9mm** | **60 ± 9mm** |
| Tayal et al., 2016 | 60 (71%) | 39 (80%) | 44 (52%) | 29 (58%) | NA | NA | **64 ± 12** | **69 ± 12** | 174 ± 17 | 178 ± 20 | NYHA III: 75%  NYHA IV: 13% | NYHA III: 62%  NYHA IV: 8% | **23 (19-29)** | **26 (23 -32)** | **199 (157-250) mL** | **151 (133-191) mL** |
| Horst et al.  2016 | **92 (69%)** | **110 (82%)** | **66 (49%)** | **82 (61%)** | 21 (16%) | 39 (29%) | **67 (60–72)** | **71 (63–75)** | NA | NA | NYHA I: 1%  NYHA II:17%  NYHAIII: 77%  NYHA IV: 5% | NYHA I: 1%  NYHA II: 10%  NYHAIII: 82%  NYHA IV: 8% | 23 ± 7 | 24 ± 7 | NA | NA |
| Lipar et al., 2016 | 35 (21%) | 21 (18%) | 94 (57%) | 66 (57%) | NA | NA | 74.3 (10.6) | 75.5 (10.1) | **147 ± 26** | **178 ± 34** | 2.3 ± 0.6 | 2.3 ± 0.6 | **26.1 ± 8.3** | **27.9 ± 9.7** | 120.2 ± 56.6 mL | 111.0 ± 52.3 mL |
| Vamos et al., 2017 | 288 (77.4%) | 139 (78.5%) | 195 (51.7%) | 103 (58.2%) | **124 (32.9%)** | **74 (41.8%)** | 66.5 ± 11.3 | 68.3 ± 10.4 | **155.3 ± 26.8** | **170.8 ± 29.8** | 2.75 ± 0.66 | 2.81 ± 0.61 | **25.3 ± 7.0** | **24.0 ± 7.9** | 66.1 ± 9.9 mm | 65.5 ± 11.4 mm |
| Cheung et al.  2017 | **328 686 (70.8%)** | **12 971 (66.3%)** | **270 191 (58.2%)** | **12 130**  **(62%)** | 293 403 (63.2%) | 12 403  (63.4%) | **70.0 ± 11.9** | **69.4 ± 13.2** | NA | NA | NA | NA | NA | NA | NA | NA |

Supplementary Figure 1a: Risk of all-cause mortality (Hazard Ratio, unadjusted) after de novo vs. upgrade CRT

**
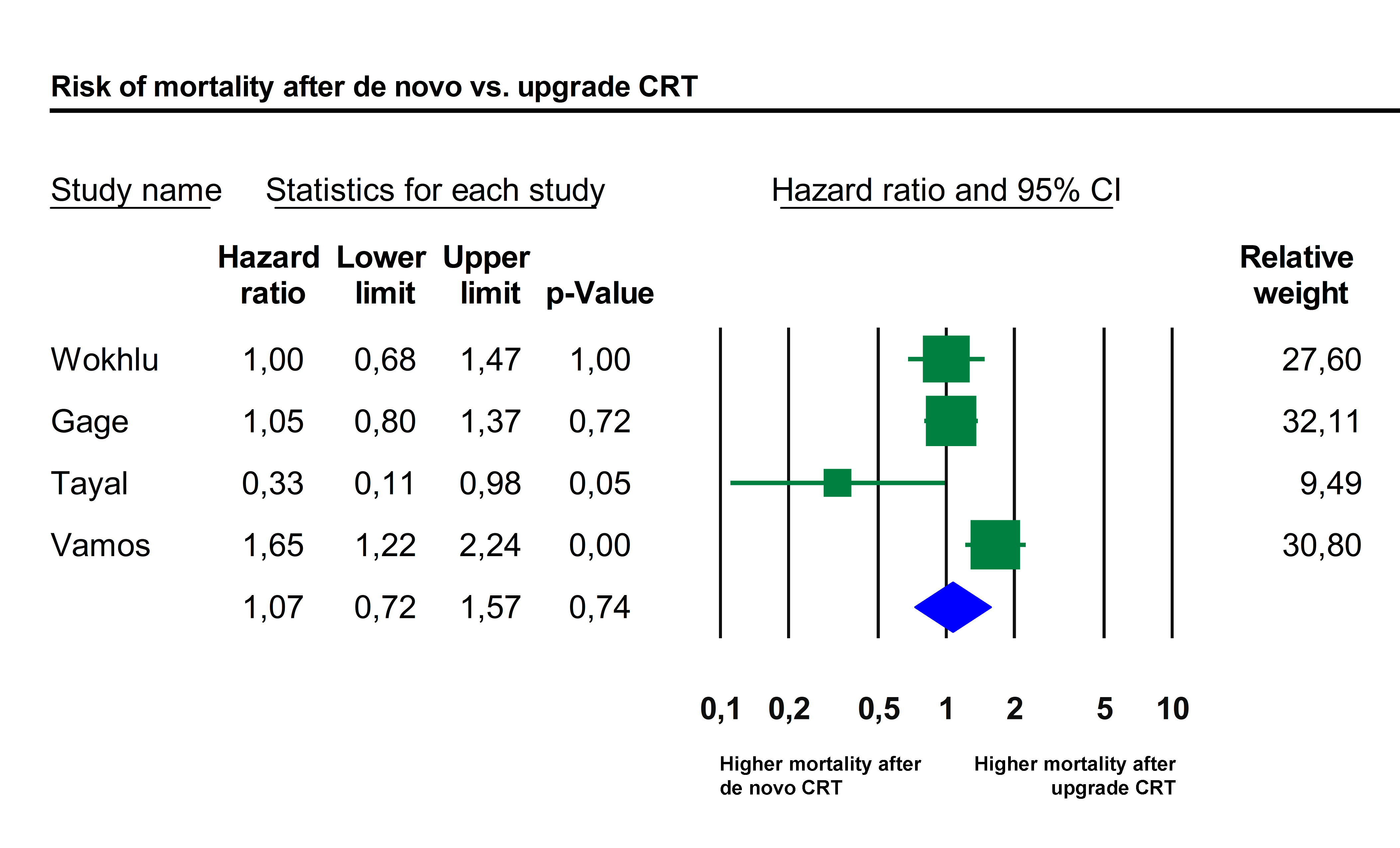
**

Supplementary Figure 1b: Risk of all-cause mortality (Hazard Ratio, adjusted) after de novo vs. upgrade CRT

**
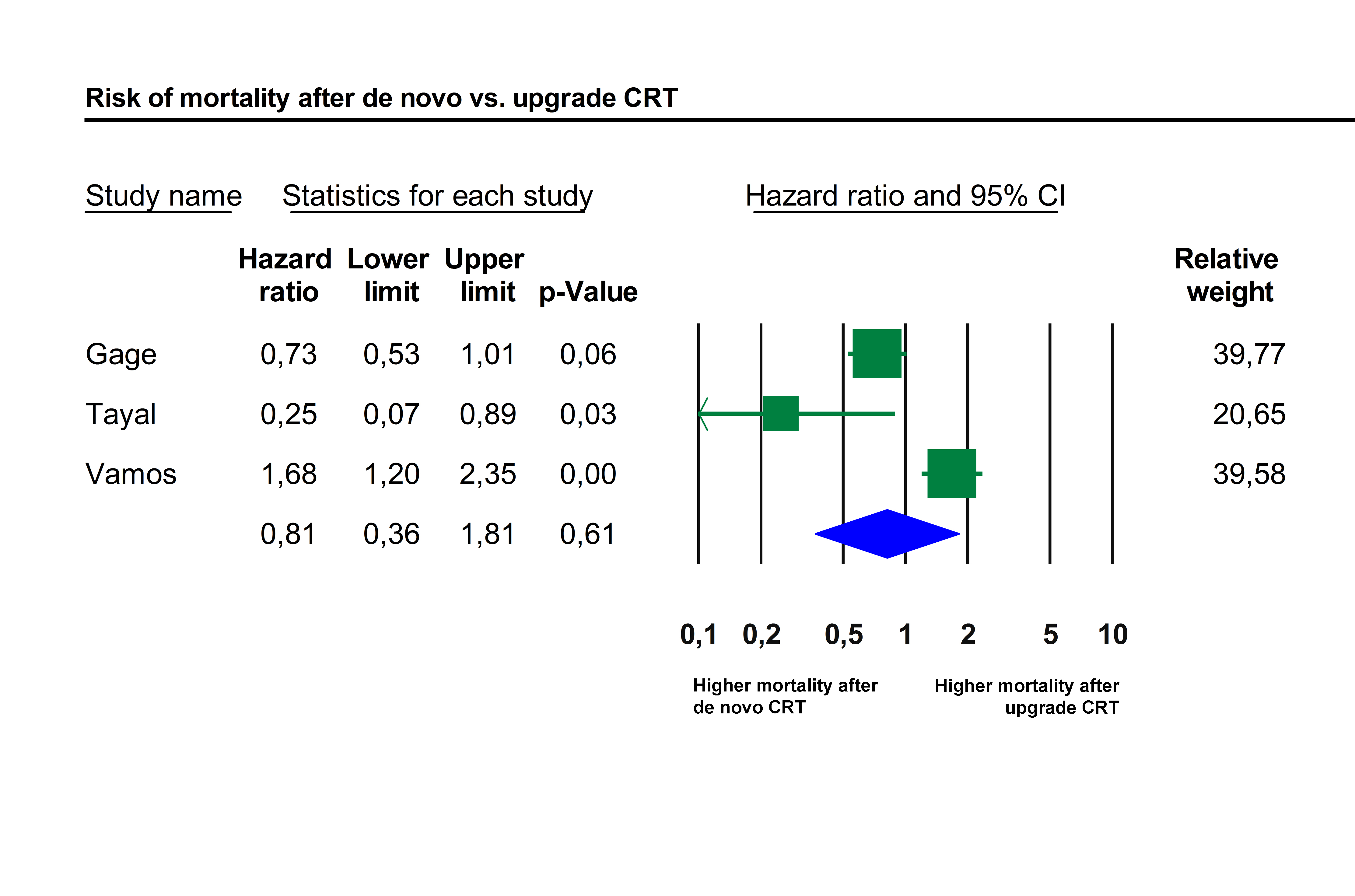
**

Supplementary Figure 1c: Risk of all-cause mortality (Risk Ratio, unadjusted) after de novo vs. upgrade CRT in prospective trials.


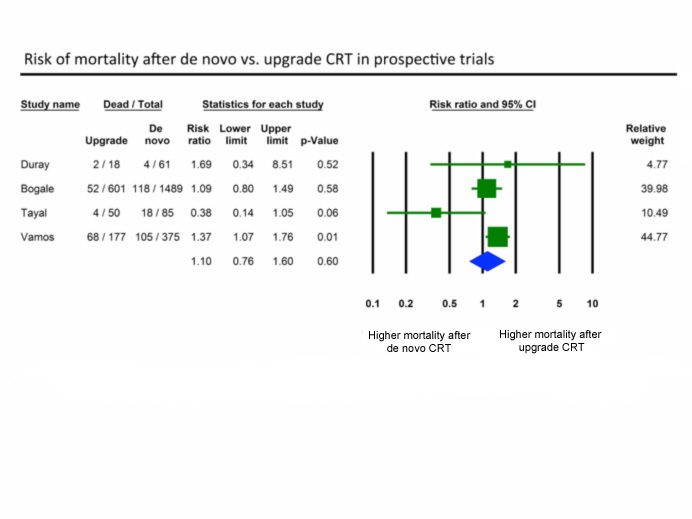


Supplementary Figure 2: Funnel plot of all-cause mortality expressed in risk ratio. Two study were trimmed to right of mean (red dot) according to the Duval and Tweedie’s trim and fill method (mortality RR: 1.186, 95% CI, 0.877 to 1.604, vs. 1.316, 95% CI, 0.983 to 1.761)


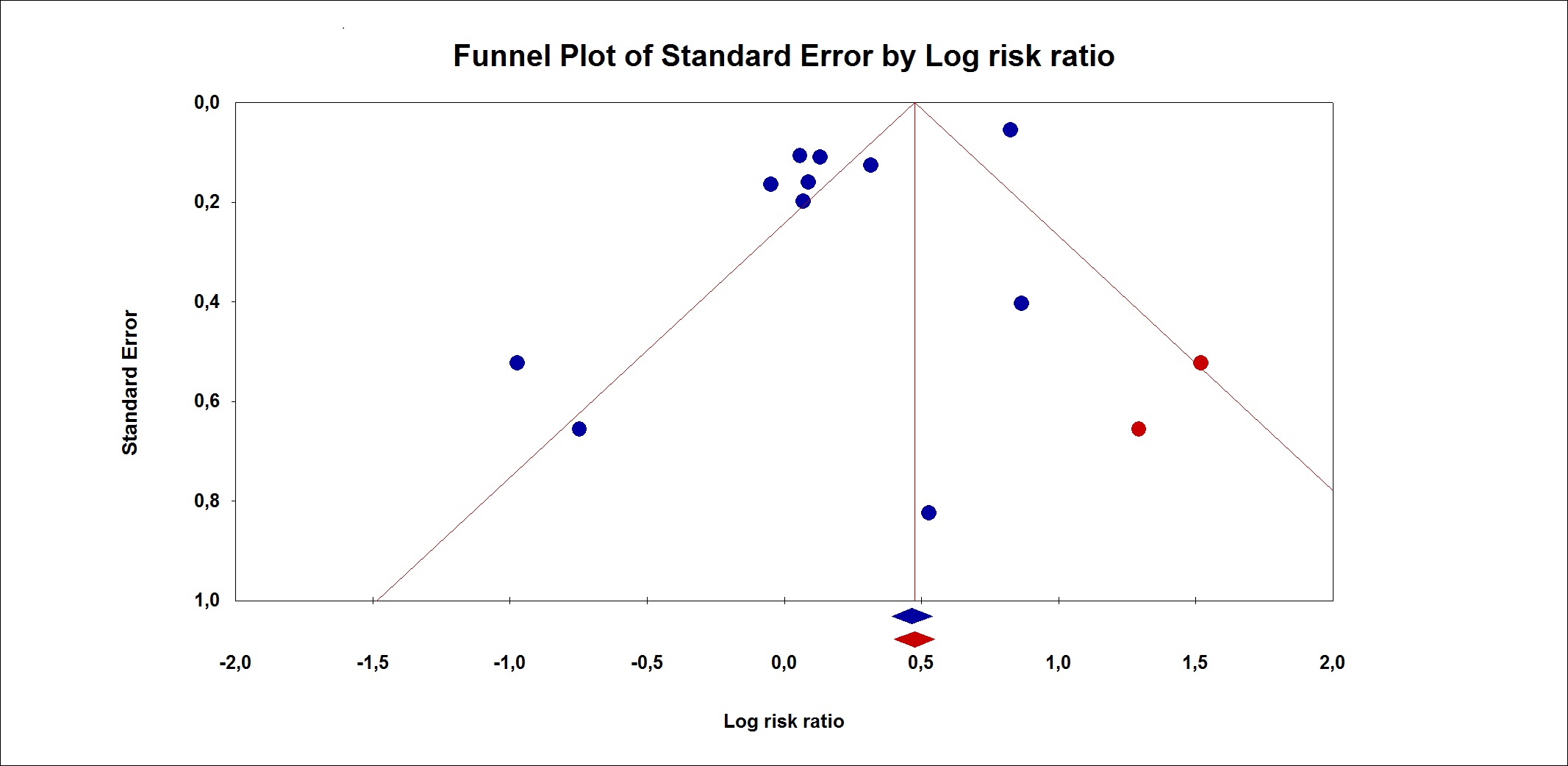


Supplementary Figure 3: Funnel plot of all-cause mortality expressed in risk ratio in prospective trials. No study should have been trimmed according to the Duval and Tweedie’s trim and fill method.


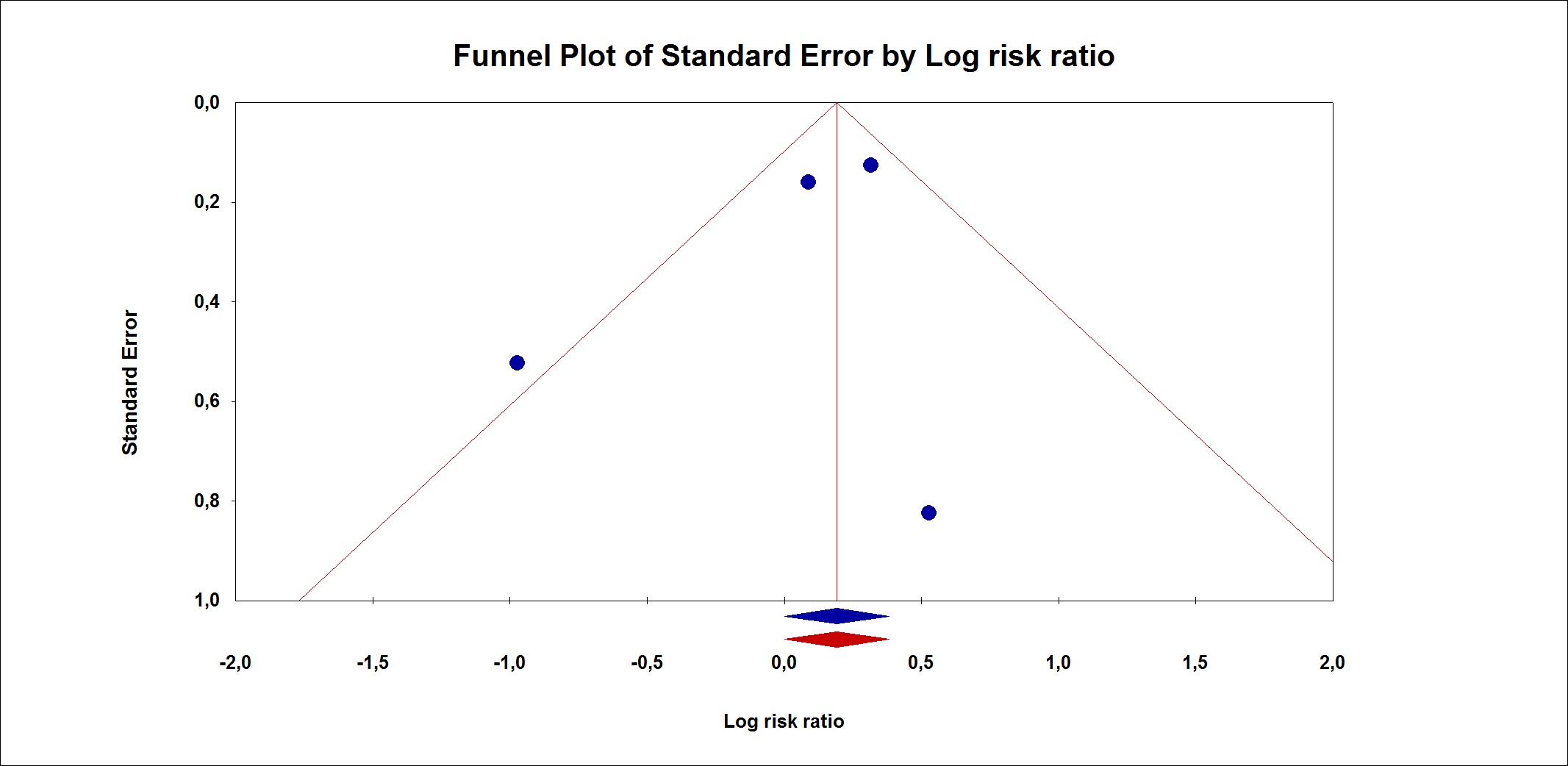


Supplementary Figure 4: Funnel plot of all-cause mortality expressed in hazard ratio (crude). One study was trimmed to right of mean (red dot) according to the Duval and Tweedie’s trim and fill method (mortality HR: 1.067, 95% CI, 0.724 to 1.571, vs. 1.206, 95% CI, 0.788 to 1.845)


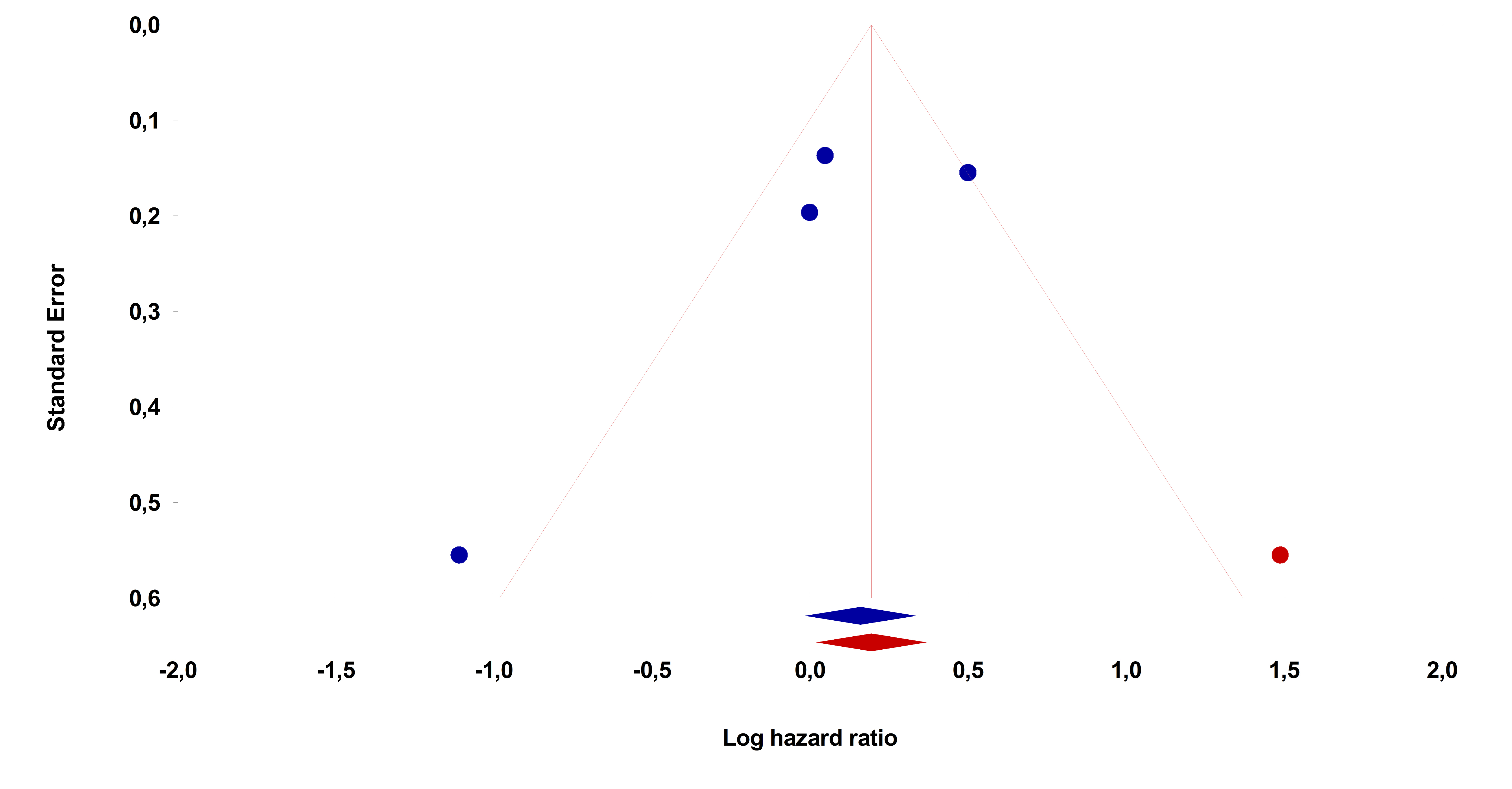


Supplementary Figure 5: Funnel plot of all-cause mortality expressed in hazard ratio (adjusted). One study was trimmed to right of mean (red dot) according to the Duval and Tweedie’s trim and fill method (mortality adjusted HR: 0.814, 95% CI, 0.365 to 1.815, vs. 1.106, 95% CI, 0.506 to 2.420)


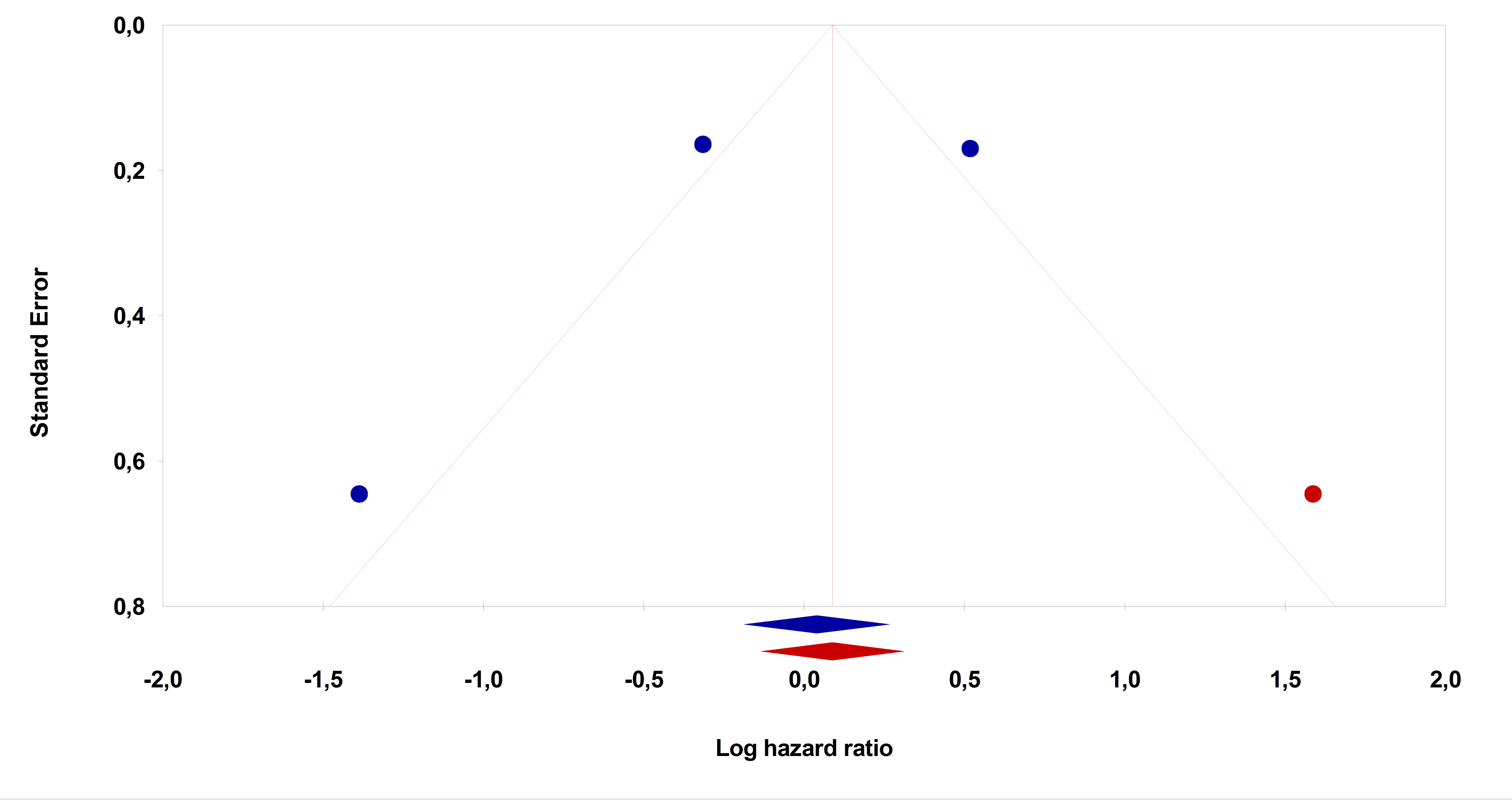


Supplementary Figure 6: Funnel plot of heart failure events expressed in risk ratio. No study should have been trimmed according to the Duval and Tweedie’s trim and fill method.


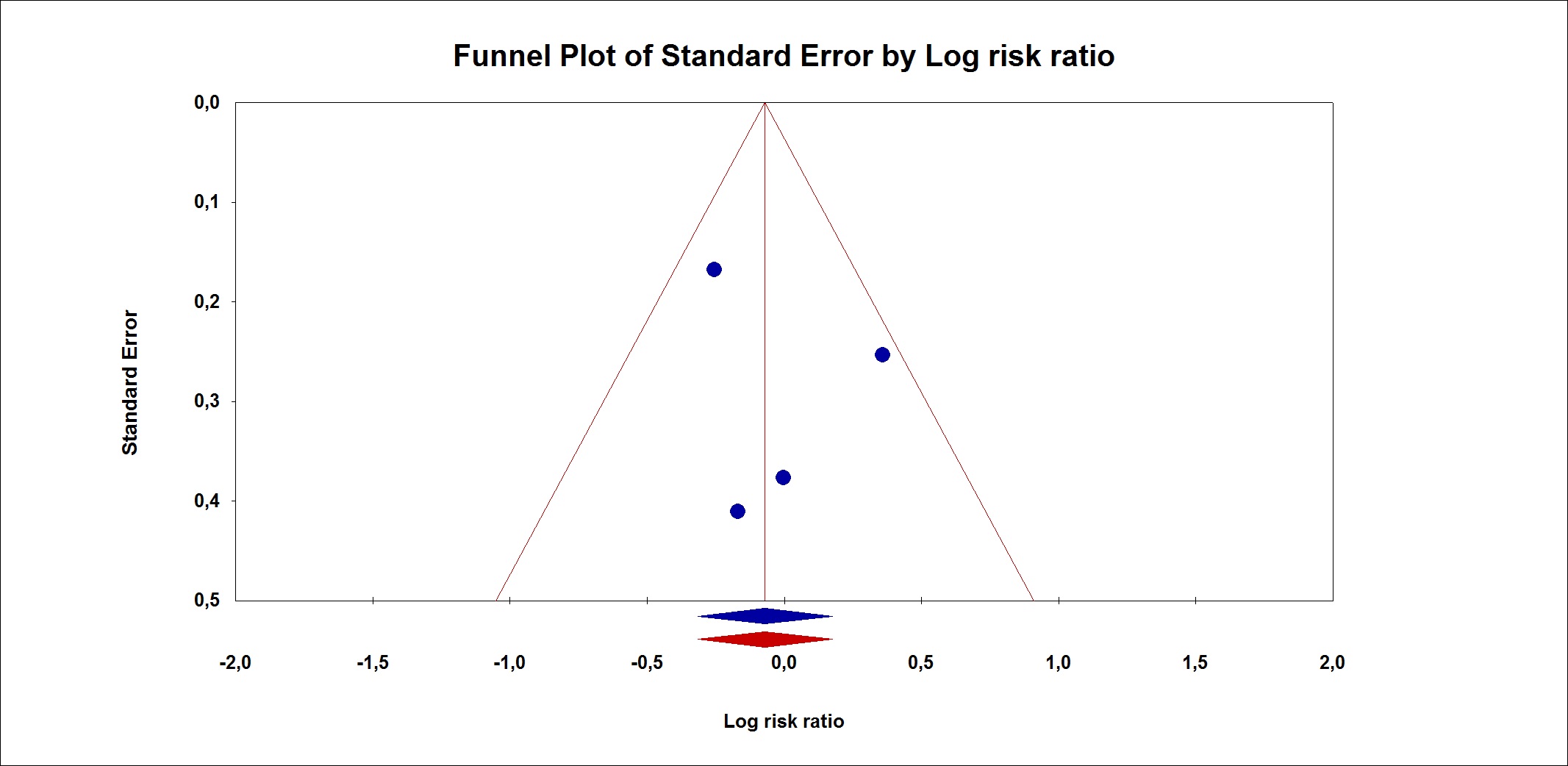

Supplement: Supplementary file 1 — (DOCX 4.53 mb) [file 10741_2017_9652_MOESM1_ESM.docx]
